# An analysis of unconscious gender bias in academic texts by means of a decision algorithm

**DOI:** 10.1371/journal.pone.0257903

**Published:** 2021-09-30

**Authors:** Pedro Orgeira-Crespo, Carla Míguez-Álvarez, Miguel Cuevas-Alonso, Elena Rivo-López

**Affiliations:** 1 Department of Mechanical Engineering, Heat Engines and Machines, and Fluids, Aerospace Area, Aerospace Engineering School, University of Vigo, Vigo, Spain; 2 Language Variation and Textual Categorization (LVTC), Philology and Translation School, University of Vigo, Vigo, Spain; 3 Faculty of Economic Sciences, Business Organization and Marketing Department, University of Vigo, Vigo, Spain; Newcastle University, UNITED KINGDOM

## Abstract

Inclusive language focuses on using the vocabulary to avoid exclusion or discrimination, specially referred to gender. The task of finding gender bias in written documents must be performed manually, and it is a time-consuming process. Consequently, studying the usage of non-inclusive language on a document, and the impact of different document properties (such as author gender, date of presentation, etc.) on how many non-inclusive instances are found, is quite difficult or even impossible for big datasets. This research analyzes the gender bias in academic texts by analyzing a study corpus of more than 12,000 million words obtained from more than one hundred thousand doctoral theses from Spanish universities. For this purpose, an automated algorithm was developed to evaluate the different characteristics of the document and look for interactions between age, year of publication, gender or the field of knowledge in which the doctoral thesis is framed. The algorithm identified information patterns using a CNN (convolutional neural network) by the creation of a vector representation of the sentences. The results showed evidence that there was a greater bias as the age of the authors increased, who were more likely to use non-inclusive terms; it was concluded that there is a greater awareness of inclusiveness in women than in men, and also that this awareness grows as the candidate is younger. The results showed evidence that the age of the authors increased discrimination, with men being more likely to use non-inclusive terms (up to an index of 23.12), showing that there is a greater awareness of inclusiveness in women than in men in all age ranges (with an average of 14.99), and also that this awareness grows as the candidate is younger (falling down to 13.07). In terms of field of knowledge, the humanities are the most biased (20.97), discarding the subgroup of Linguistics, which has the least bias at all levels (9.90), and the field of science and engineering, which also have the least influence (13.46). Those results support the assumption that the bias in academic texts (doctoral theses) is due to unconscious issues: otherwise, it would not depend on the field, age, gender, and would occur in any field in the same proportion. The innovation provided by this research lies mainly in the ability to detect, within a textual document in Spanish, whether the use of language can be considered non-inclusive, based on a CNN that has been trained in the context of the doctoral thesis. A significant number of documents have been used, using all accessible doctoral theses from Spanish universities of the last 40 years; this dataset is only manageable by data mining systems, so that the training allows identifying the terms within the context effectively and compiling them in a novel dictionary of non-inclusive terms.

## Introduction

The frequent problem of gender bias has generated a considerable amount of research (as will be seen in later paragraphs). However, there is little research on the phenomenon of second-generation gender bias. This bias occurs when a person continues to unconsciously make stereotype-based evaluations but consciously rejects "gender stereotypes" ([1,2]). This is possible because this indeterminate number of people unconsciously continue to make evaluations based on stereotypes derived from traditions, life experience, norms and/or culture. Academic texts and in particular doctoral theses, we argue, are overwhelmingly in this situation. The very object of the thesis itself, its development, and its expression in a document (in the case of having gender biases), can be considered to be due to involuntary and automatic mental associations based on gender.

Currently, research on unconscious bias has focused mainly on labor issues or workplace organization [3–5] or on barriers to women’s leadership ([2,6–10]).

Unconscious bias can be revealed by analyzing data patterns and identifying gender gaps, but doing so in large volumes of data can be a herculean or unmanageable task. With the development of artificial intelligence, it has been possible to process large volumes of data that can address various research domains. There are still certain difficulties present: for example, if that data is loaded with gender-stereotyped concepts, the resulting application of the technology will maintain this bias. Currently, the use of inclusive language is one of the initiatives that try to guarantee equal treatment for all people. This work develops an algorithm for the detection of the non-inclusive use of the Spanish language in the field of academic texts, using machine-learning techniques. Language is one of the areas where discrimination is observed due to the lack of equal treatment of women and men. Many of the debates in artificial intelligence on the subject of gender bias reflect those related to gender equality in society since the 1970s.

Different studies and organizations have studied or even developed user-friendly toolkits or guides with recommendations on language bias [11–13]. Other studies have focused on different aspects of gender and language, mainly on the differences in the way women and men speak as well as the structural and functional aspects of gender-related variation and change in individual languages [14–16]. Different comparative analyses of gender in different languages [17] have been developed, and a sociolinguistic analysis of several languages of different morphological characteristics [18].

Text mining (that describes procedures for obtaining key information from a text by detecting hidden patterns in unstructured documents), has been under research over the last years for many different areas [19–22]. The studies of the use of inclusive language in a document might be considered similar to automatic sentimental classification: opinion mining tries to extract opinion, which are subjective from expressions, and the detection of non-inclusive language is about finding whether the use of the same terms is considered as inclusive or not [23–25]. Most of the investigation lines for text classification were based on extracting algorithms from samples set to train algorithms, including support vector machine (SVM) [26–28], k-nearest neighbor (kNN) [29,30], Naïve Bayes (NB) [31–34], CNN [35] decision tree [36–39], inside the classification paradigm. Big data analysis using different flavors of artificial intelligence has also shown interesting results in many other areas ranging DNA studies [40,41], vehicle theft identification [42], intrusion detection [43,44] and more. This research analyzes the possible unconscious gender bias in a study corpus consisting of more than one hundred thousand doctoral theses, written in the Spanish universities. For this purpose, an automated algorithm was developed to evaluate the different characteristics of the document and to search for interactions according to age, year of publication, gender and field of knowledge in which the doctoral thesis is framed. The main contributions of this work are: a) The algorithm that detects non-inclusive usages of the language within an Spanish text (given a generic text, every word in every sentence is evaluated and qualified as non-inclusive usage or not); b) The study along over one hundred thousand doctoral theses (the whole Spanish production in electronic format) to find out, according to the developed algorithm, how different properties of the document (author’s age, gender, year of publication) affect the non-inclusiveness index of the doctoral thesis. The paper is structured as follows: section 2 presents the background of previous work provided by the literature, and how the problem was approached; section 3 explains the proposed methodology followed during this research, including the automated algorithm; section 4 display and analyzes the obtained experimental results; finally, section 5 details the conclusions and future work.

## Background and related work

Constraints to the description of gender have been explored across languages or countries [45], as in the case of English [46], German [47], Swedish [48], Chinese [49–51], Polish [52], Italian [53,54] or French [55]. In this line and with respect of the Spanish language, there have also been several research studies [56–58]. For the case of academic texts, [59] analyzed documents from a university to detect the presence of a neutral and sex-linked language as a measure of the impact of regulations on language change. They studied documents between 1969–1972 and 1978–1979. The newer documents were found to contain significantly fewer sex-linked language elements and markedly more neutral elements than the older documents. Most of the changes involved language structures that had received considerable public debate.

When working with large amounts of data, it is necessary to resort to machine learning techniques. For the general classification problem in text mining, a number of classification techniques are explored: [60–63] combined several to obtain an adequate categorization. The support vector machine has historically been tested as a solution for binary classification in text environments under different approaches [64].

An algorithm can also be biased by gender, ethnicity, nationality, wealth or other social characteristic if it performs differently or with different levels of efficiency for different groups. A common issue found through literature review is the problem of the bias that word embeddings suffer from, according to the text corpora the different solutions are built with. The subject has been studied and some research suggest that gender bias has not been solved yet [65]. Automated detection of a use of specific language has been already covered in previous research: [66] deduce the meaning of words by associating them with other words that tend to occur in the same document. Using this approach can lead to conservative implications, such as that “homemaker” is part of the common meanings of the word “woman”, and that “programmer” is part of the meaning of the term “man” [67].

Several attempts to eliminate that favoritism in the context of gender are present in the literature, including those that approach the problem with a similar point of view as our research [68]. In [69], unintended bias is addressed from the perspective of detecting misogyny; in [67], authors claim to remove gender stereotypes while keeping reasonable and natural embeddings; sexist messages are also studied [70] or any offensive language detection [71].

This discrimination is increasingly manifested on social media: [72] examines the problem of gender discrimination and attempts to move beyond the typical surface-level text classification approach by identifying differences between genders in the way they use the same words. [73] presented several configurations of a language-independent classifier for predicting the gender of Twitter users. [74] proposed a set of criteria that a tweet should exhibit in order to be classified as offensive. [75] covered detecting hate speech and proposed novel methods that take advantage of knowledge-based generalizations for bias-free learning, using Wikipedia and Twitter as the dataset. [76] finds that racist and homophobic tweets are more likely to be classified as hate speech, but that sexist tweets are generally classified as offensive. Tweets without explicit hate keywords are also harder to rank for. [77] also studies hate speech on Facebook. [78] proposes the application of text mining techniques to online gender discourse through the analysis of shared reviews in electronic word-of-mouth communities (eWOM).

[67] shows that even word embeddings trained on Google News articles exhibit female/male gender stereotypes to a disturbing extent. They quantitatively demonstrated that word-embeddings contain biases in their geometry that reflect gender stereotypes present in broader society. Due to their wide-spread usage as basic features, word embeddings not only reflect such stereotypes but also can amplify them. This poses a significant risk and challenge for machine learning and its applications. All these approaches consider only English embeddings. A few recent studies focus on measuring and reducing gender bias in contextualized word embeddings [79–82]. [83] mitigates bias by saving one dimension of the word vector for gender. [84] proposes a regularization loss term for word level language models. Moreover, [65] shows that mitigation methods based on gender directions are not sufficient, since the embeddings of socially biased words still cluster together.

[85] work lends itself to a computational approach to identify gender bias and could be used to remove it from training data for a machine learning algorithm. Similarly, Facebook uses algorithms to select content for users’ newsfeeds [86], and combines lexical-based and machine-learning techniques. The results obtained through this approach show that it is feasible to perform sentiment analysis in Facebook with high accuracy (83.27%) [87].

Albeit an important part of the previous research is focused on the English language, there are as well some minor attempts to address the issue in different languages such as French and Spanish [55,56]. That research explains how word embeddings experiment bias when it comes to gender analysis (and also when looking for hate in speech); they as well show certain methods to tackle that problem.

Table 1 summarizes the most relevant previous works in this area, shown in this section:

**Table 1:** Previous works comparative table.

Research	Reference	Contribution
Constraints to the description of gender	45	Generic to every language
	55–58	Spanish specific
	46–55	Other languages
Gender bias	65	Studied the issue, but no solution was given
	66	Attempt to eliminate favoritism
	67, 79–87	Studied why gender bias happen
	69	Misogyny detection
	70	Analysis of sexist messages
	71	Sex offensive language
	72–78	Hate

The research hypotheses are as follows:

- H1: The development of an automatic text extraction algorithm would help to analyze gender bias.
- H2. The use of inclusive terms is directly related to age.
- H3: The use of inclusive terms is related to the field of knowledge.
- H4: The use of inclusive terms is directly related to gender, being lower in women.
- H5: Unconscious bias is a characteristic of academic texts, particularly doctoral theses.

## Proposed method

### Data

The context for this research is the corpora of doctoral theses wrote in Spain since 1974, when the first digital treatable documents are found. Table 2 summarizes the key indicators for this process as regards the amount of text to be treated:

**Table 2:** Key indicators.

Indicator	Value
Number of thesis per year (average)	2232
Number of pages per thesis (average)	301
Number of words per thesis (average)	124,012
Publication range	1974–2020

Although the total number of theses found were over 257,000, only 100,450 could be used (the other ones were either not public domain or not in a legible file).

**Fig 1:**
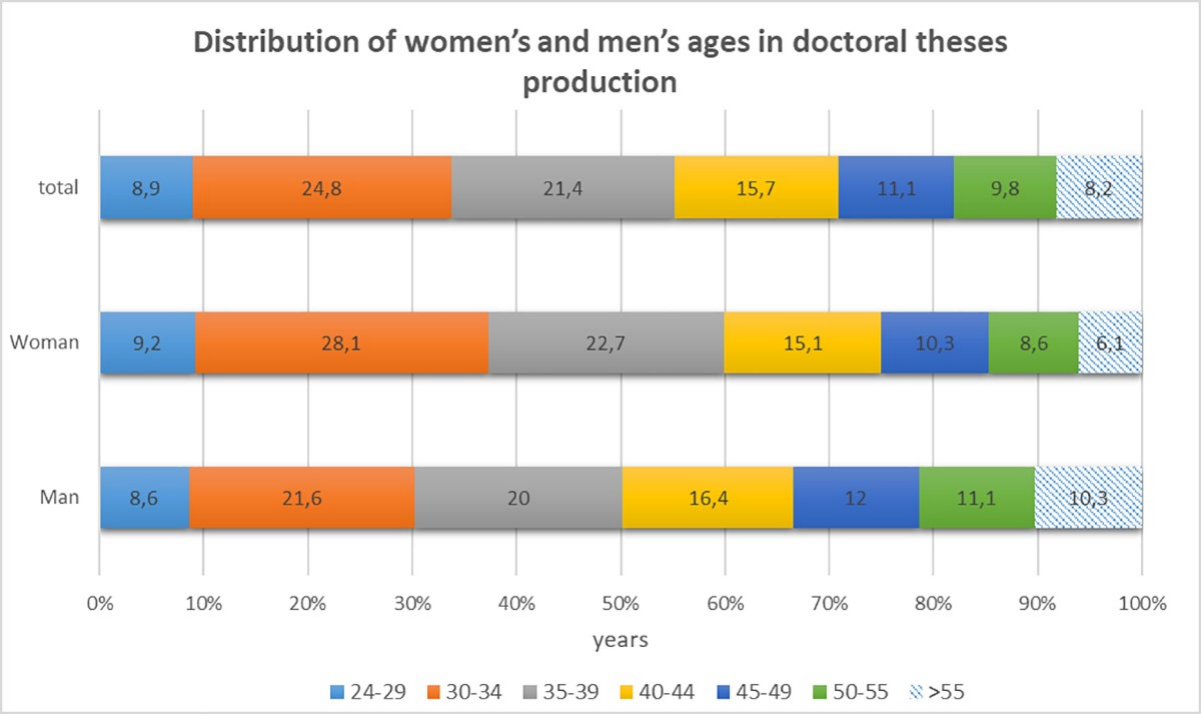
Author’s age and gender distribution.

The first area of interest was the age of the author, and how it could affect the use of inclusive language within his/her doctoral thesis. The age and gender of the authors are show in Fig 1.

The documents were created along 46 years at the Spanish universities. Only those that were public domain and obtained as PDF files were treated. In this data corpus, over 12 billion words were found using over 9000 different elements, being, therefore, a very complete representation of the Spanish language.

Another important aspect is that every document in the data set belongs to one of the UNESCO categories for doctoral thesis, as displayed on Table 3:

**Table 3:** Categorization of doctoral theses.

Category	Theses (%)
Medical sciences	14.6
Life sciences	10.3
Technologic sciences	9.6
Chemistry	6.8
History	6.1
Economical	5.5
Math	5.5
Physics	5.1
Psychology	5.0
Art	4.9
Sociology	3.4
Legal sciences	3.3
Pedagogy	3.2
Agricultural sciences	3.1
Linguistics	2.9
Earth and space sciences	2.8
Political sciences	2.5
Philosophy	1.2
Geography	0.7
Demography	0.6
Astronomy and astrophysics	0.5
Ethics	0.4
Logic	0.3

**Fig 2:**
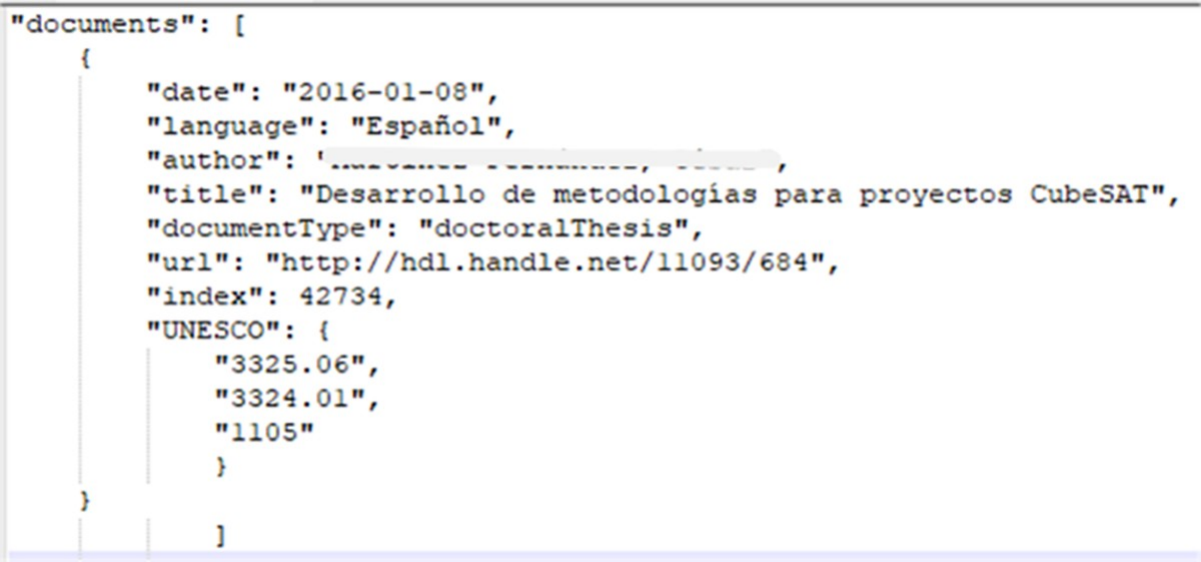
Stored document metadata.

A deep learning model was the option chosen due to the need to provide the algorithm with information on a small set of training documents (what should or should not be inclusive for a term in context), and subsequently label terms in different contexts to validate the model. Metadata were also obtained that described the document: the date of publication, the language in which it was written, the name of the author, the title of the research, and the UNESCO code that characterizes the area of knowledge; that information was obtained, structured, and stored in a database, as depicted in Fig 2:

### Algorithm design

**Fig 3:**
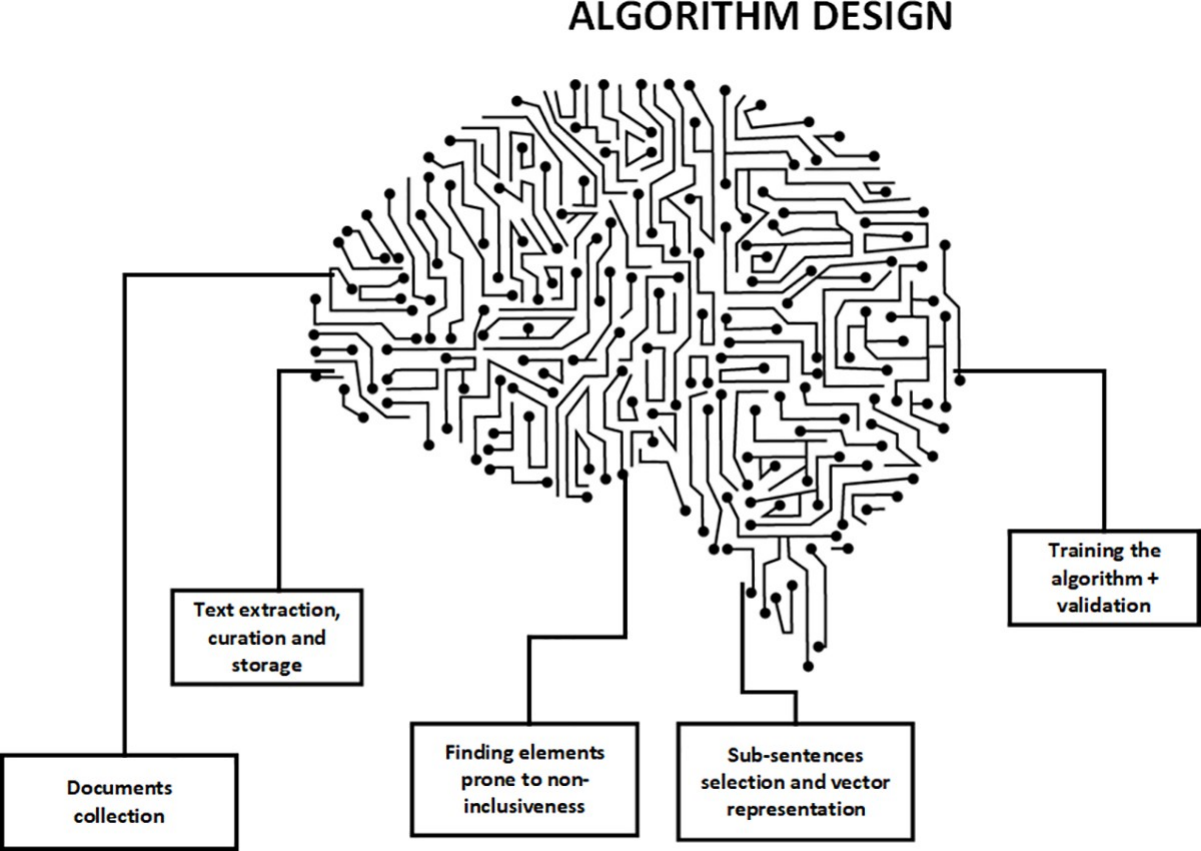
Conceptual definition of the algorithm design.

Convolutional Neural Networks (CNN) are well known in the area of text classification [88]. Their capacity to learn from sentences having the same or similar words in different parts of the phrase allows us to capture relevant information. Anyway, the architecture of the CNN needs to be adapted to the specific problem to be solved, and also a future work should be to analyze whether the generated embeddings depend on the domain of the corpus or not. Much research has been explored in text mining and NLP, as well as the world of text classification. The whole process is summarized in Fig 3:

#### Document collection

The documents that make up the corpus were obtained from the universities that make up the Spanish university system, hosted on their official servers. In the first place, the raw documents were obtained, to later process them and extract from them the information related to authorship, date, subject, etc.

**Fig 4:**
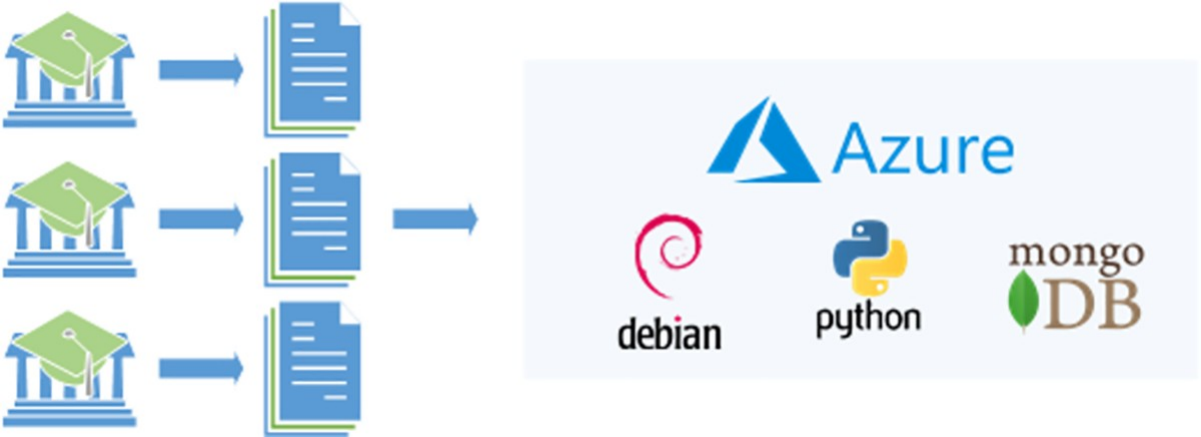
Technological stack.

An ecosystem of virtual machines has provided the IT infrastructure capabilities: hosted in Azure, it consisted of Debian MVs on which Python scripts were executed, and hosted the information in SQL-Server and MongoDB databases. Fig 4 describes that infrastructure:

#### Text extraction, curation and storage

Once the raw text of each document has been extracted, it has been processed to prepare it to be analyzed by an automatic algorithm. Special emphasis has been placed on eliminating those words that, belonging to areas of the document where studying its inclusiveness does not really apply, could negatively impact the process without providing information: mathematical formulae, portions of texts in other languages within the text in the Spanish language, page numbers, section names, table headers, etc.

This process has been carried out using different libraries oriented to NLP (natural language processing), both on Python and in C # using.Net Core.

#### Data obtention

Once the information was available as extracted text, properly structured, and stored, the next step was to provide a numerical representation to the text. The first focus was to locate all those adjectives and common nouns that, present in the text in their masculine form, also have a valid feminine form in Spanish (for example: “profesor” -teacher-, which has its feminine form “profesora”-female teacher-, valid). Common nouns and adjectives are those that, being able to exist in their masculine, feminine, or neutral form, are key to determine whether their use might be considered non-inclusive. Accordingly, the Freeling library (specialized in morphologic analysis) was used to provide noun and adjective detection; in this case, the hmm_tagger (Hidden Markov Model PoS Tagger) was employed to provide PoS (part of speech) on the text, implementing a classical trigram Markovian tagger. Tables 4 and 5 display the characteristics of the pairs of attribute/value for common nouns and adjectives:

**Table 4:** Noun tags.

Position	Attribute	Value
0	Category	N
1	Type	C (common), P (proper)
2	Gender	M (masc.), F (fem.), C (common)
3	Number	P (plural), S (singular)
4	Class	N/A
5	Subclass	N/A

**Table 5:** Adjective tags.

Position	Attribute	Value
0	Category	A
1	Type	P (possessive), O (ordinal), Q (qualificative)
2	Degree	N/A
3	Gender	M (masc.), F (fem.), C (common)
4	Number	P (plural), S (singular)
5	Person	N/A
6	Number of possessed	N/A

Under this methodology, the whole corpus could be analyzed putting special emphasis on the search of parts of sentences where common nouns and adjectives could be found under their masculine form, ruling out those that do not have a feminine form in the Spanish dictionary, and creating parts of sentences to be evaluated. Since sentences need to have a context to be properly analyzed, the algorithm looked for their beginning, using a punctuation mark (comma, period, semi-colon, etc.); that punctuation mark became the beginning of a new sub-sentence to be isolated and stored. In that new sub-sentence, the first instance of a common noun or adjective in their masculine form was searched for, until the next punctuation mark (if during this process no element could be found, the sub-sentence was discarded). Once an element was found, the next punctuation mark was looked for; after several iterations, it was determined that the subsentence should not contain more than 7 other words before the next punctuation mark was found, counting from the element that was discovered: that provided meaningful sub-sentences that could be properly analyzed and characterized, in the next step.

#### Vector representation

The goal of this step was to generate a sequence of word embeddings using the input acquired in the previous step, to finally obtain a numerical representation of the relevant sub-sentences that contained potential non-inclusive usages of the elements found within the text. The idea behind this approach is that it is the context where a masculine common noun or adjective is used, that give rise to its non-inclusiveness (not the term on its own). Therefore, every sub-sentence was characterized with a sequence of vectors, where each vector was calculated as shown in Table 6:

**Table 6:** Mapping vectors to words.

Field	Name	Values
1	ID	Auto incremented value, unique for word within the Spanish dictionary
2	Category	1 (adjective), 2 (common noun), 3 (proper noun)
3	Position	Position within the relevant context
4	Distance	Distance in words to the first noun/adjective element in the sub-sentence
5	Gender	1 (feminine), 2 (masculine)
6	Number	1 (singular), 2 (plural)
7	Occurrences in document	Number of times the element is present within the document
8	Total occurrences	Number of times the element is present in the whole dataset

#### Architectural model and algorithm training

**Fig 5:**
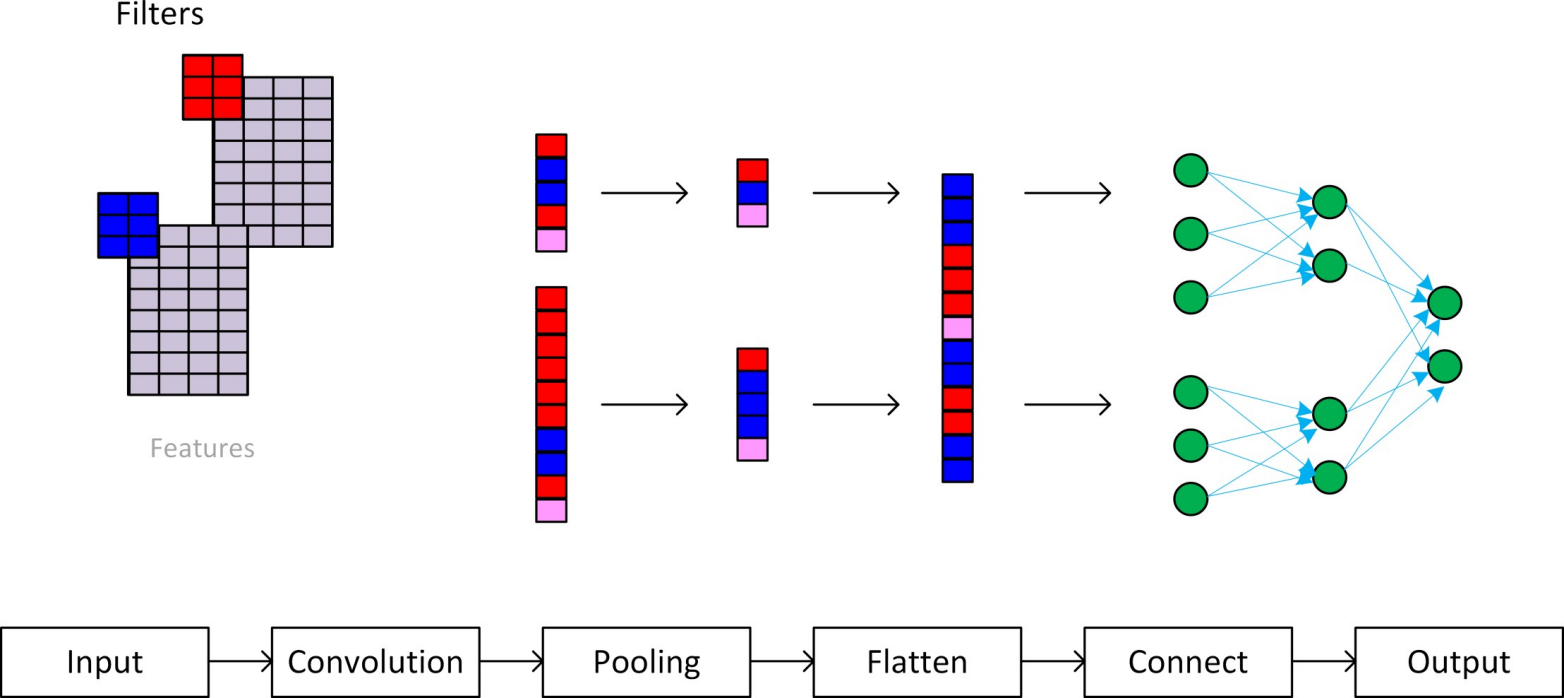
Architecture of the model of the CNN.

Fig 5 depicts the architectural model of the neural network developed to address the classification. In the previous step we had achieved an 8-dimension vector of every element of the relevant sub-sentences found in each document. Each sub-sentence of q words is, consequently, represented as a matrix:

$$P\;\epsilon \;R^{q\;x\;8}$$ (1)

The architecture is based on a CNN model, to provide classification to determine when each sub-sentence can be considered as non-inclusive use of the language or not. After experimenting with the data iterating through different methods, we empirically determined (after a tenfold cross validation) that every convolution operation to be applied would be implemented as 128 filters; we decided to use a pooling layer based on max pooling, and a final perceptron with two layers. Fig 5 displays the architecture:

Data labelling was performed by a group of experts on the field, which were fed with 8,200 sub-sentence samples and their extra context (validating, as well, the size of the sub-sentence for relevant non-inclusive identification). Every sub-sentence was reviewed by two different persons, providing a binary classification (“Inclusive”/”Non inclusive” usage). The process was accomplished in two rounds, where the second was to categorize only the sub-sentences that had obtained different classification; finally, that third vote provided the final categorization for the sub-sentence.

Out of the labelled samples, 6,550 were used to train the algorithm, and 1,640 were kept for validation purposes, in a standard 80/20 ratio.

#### Validation

A confusion matrix is a very common description of the performance of categorization models. The matrix (indeed, a table) displays the number of accurately and erroneously classified samples, compared to the actual outcomes (target value) in the test data. In the field of artificial intelligence and especially in the problem of statistical classification, a confusion matrix is a tool that allows the visualization of the performance of an algorithm that is used in supervised learning [89].

As well, certain indicators were also added to cope with the common issue binary classification comes with: false negatives (any erroneously identification by the algorithm of a sub-sentence as non-inclusive usage, when the truth is that the usage was inclusive), are more harmful than false positives. When the panel of experts reviewed an undetected non-inclusive, they felt more permissive than when the algorithm claims a non-inclusive usage for a proper language utilization.

Table 7 shows the notation that was used:

**Table 7:** Performance parameters.

Indicator	Value
Indicator	14.6
Number of sub-sentences	10.3
Number of true non-incluisve sub-sentences	9.6
Number of true inclusive sub-sentences	6.8
False positives	5.5

## Results and analysis

The analysis was divided in two parts: first, testing the reliability of the algorithm to determine the inclusive usage of language in a part of speech (subphrase); second, testing on a large number of documents, in this case Spanish theses, to draw conclusions about bias.

### Algorithm

To evaluate the performance of the algorithm, several data from the labelled dataset was kept for validation (a 20% split). The results calculated by the algorithm were compared to those obtained by the panel of linguistics, and compared using well-known standard indicators; Table 8 displays the scores obtained:

**Table 8:** Performance of the algorithm.

Variable	Value (%)
Error	5.0610
Accuracy	94.9390
False positive ratio	0.8505
False negative ratio	31.0044
False positives	12
False negatives	71
Validation labeled samples	1640

The results can be considered as quite reasonable. The accuracy is over 94%, where a certain level of misclassification was expected. The false negative ratio is over 30%, which may be reasoned as cases where a human determines a truly non-inclusive usage and the algorithm could not tell; the false positive ratio stays well below 1%, matching the expectations at the beginning of the project. Moreover, the false positive ratio kept well under the false negative ratio: in a non-inclusive classification problem, the fact of finding a misclassification by an algorithm is not positive at all, but much worse is the case where a false positive occurs, than a false negative: a false positive is assumed as something that should have been detected, but that the algorithm did not perceive; a false negative is perceived as a less acceptable failure, since it generates greater mistrust by having considered a text that was not inclusive as non-inclusive. In our case, as the FPR (false positive ratio) remains below the FNR (false negative ratio), and both are relatively low, the final result is considered positive.

**Fig 6:**
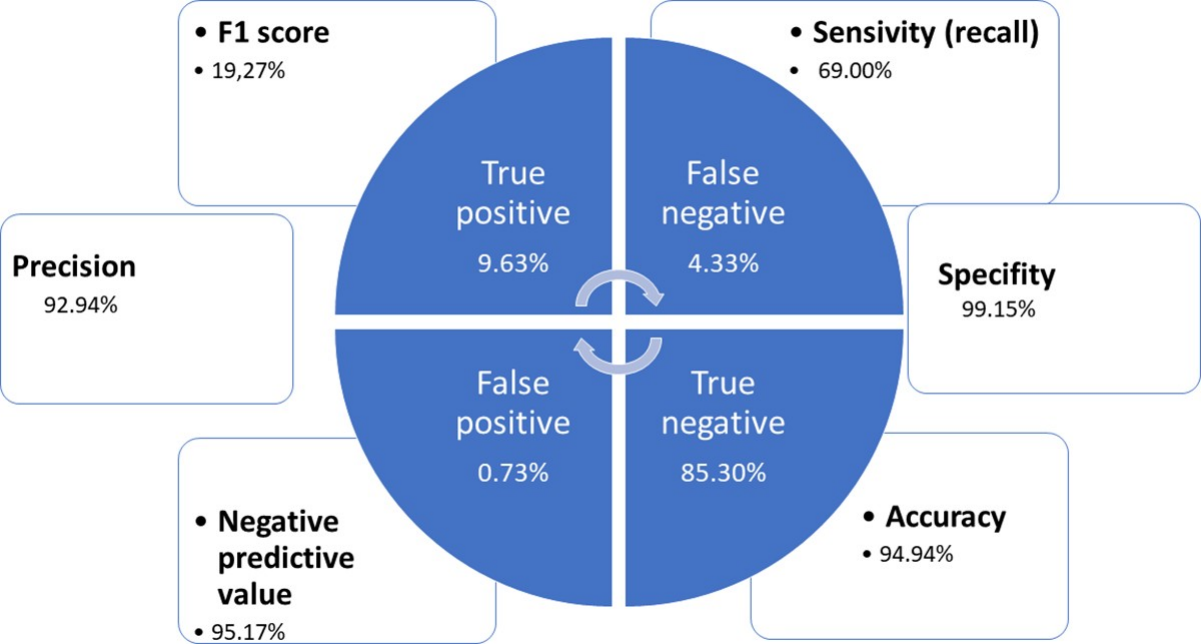
Confusion matrix.

We may find in Fig 6 the confusion matrix for the obtained values, showing quite reasonable results as for the performance of the algorithm:

### Dataset

An index of inclusiveness was defined for every document, as the ratio between the number of non-inclusive usages were found in a text, for every thousand words:

$$I_{document}=\frac{Non-inclusive\;instances}{\frac{Number\;of\;words\;in\;the\;document}{1000}}$$ (2)

Every document was characterized by its index and, consequently, different conclusions arise studying the evolution of the value of the index according to different origin regions, age of the author, and evolution throughout time.

Referring to Fig 1, the distribution of the average number of non-inclusive terms per document according to the age of the author and its gender is shown in Table 9:

**Table 9:** Age of the author, and impact on non-inclusiveness.

Age	Men	Women	Average	Deviation
24–29	14.29	13.07	13.68	0.61
30–34	15.31	13.46	14.38	0.93
35–39	14.82	13.58	14.20	0.62
40–44	15.65	14.16	14.90	0.75
45–49	16.62	14.62	15.62	1.00
50–55	21.41	16.79	19.10	2.32
>55	23.12	19.24	21.18	1.94
Average	17.32	14.99		
Deviation	3.23	2.08		

Considering the average for each age group, it can be seen that the highest mean score corresponds to the oldest group (> 55 years) while the lowest mean score would be that of the youngest group (24–29 years) regardless of gender. Based on these results, it can be said that there is a direct relationship between age and the use of inclusive terms in the texts. The most important gap happens after the age of 50, both in the average divergence with the previous bands and in gender discrimination.

It is also important to note that the older the age group, the more marked the gender difference between men and women is, also decreasing with the age of the group. It also turns out that the youngest have the lowest incidence. It is true in all cases that the male gender continues to use more non-inclusive terms than the female gender, regardless of the age group that we consider. In the same vein, the results would indicate a significantly higher support for inclusive language on the side of the female participants compared to the male ones.

**Fig 7:**
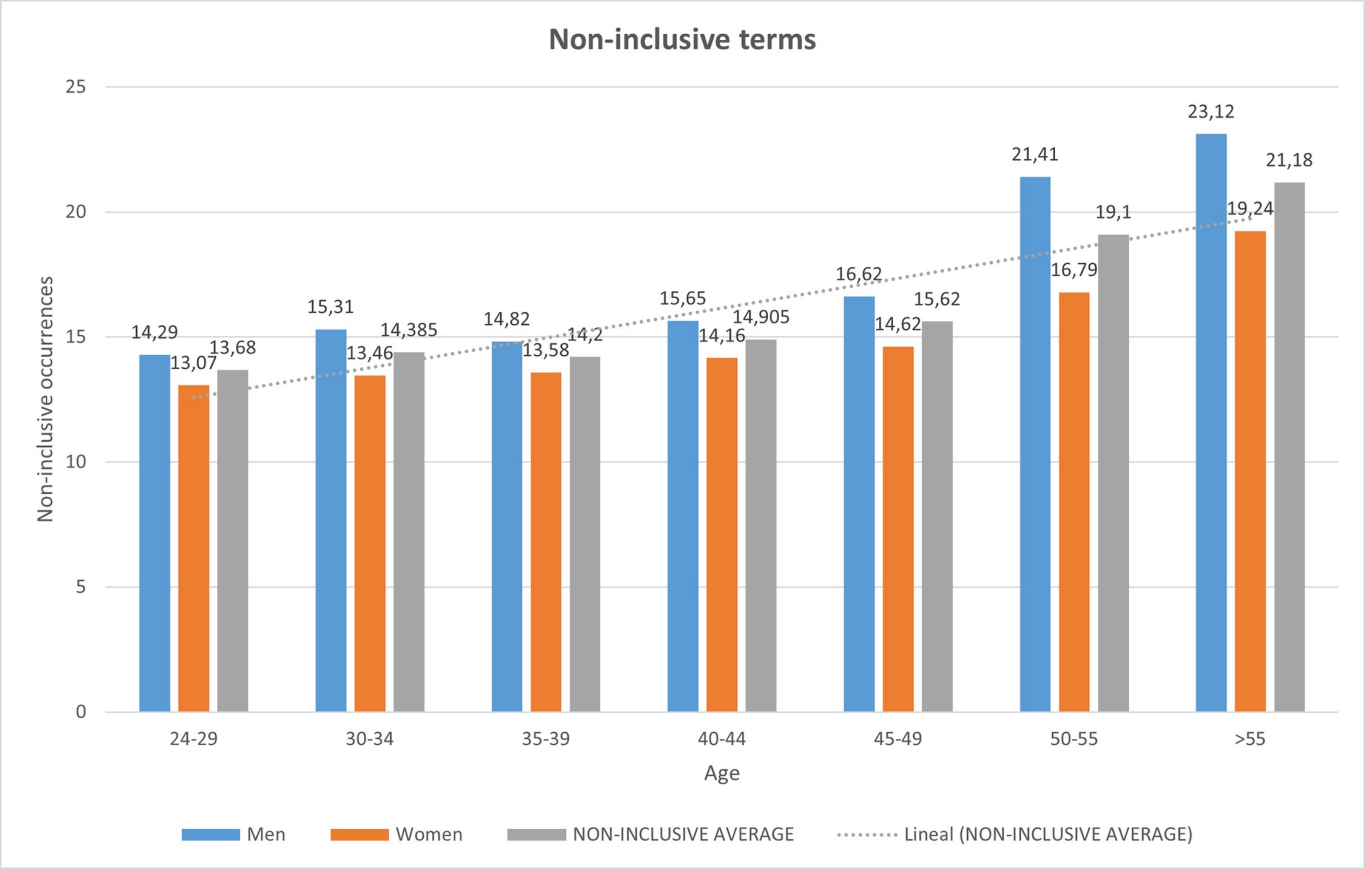
Age of the author and the impact on the non-inclusiveness.

Fig 7 displays the non-inclusiveness occurrences according to the age of the author. As expected, the youngest people are the ones who had the least use of non-inclusive terms, which means that current social conditions are changing the perception of their use.

These results are in line with those obtained in other investigations that point to the existence of a direct relationship between age and the use of inclusive terms [90–93]. It was not found in the literature that at a younger age, opinions towards inclusive language were more polarized according to gender [94]. This also has its explanation in the type of research carried out focused on doctoral theses, which results in that in the case of bias, it should be unconsciously [85–88,95].

**Fig 8:**
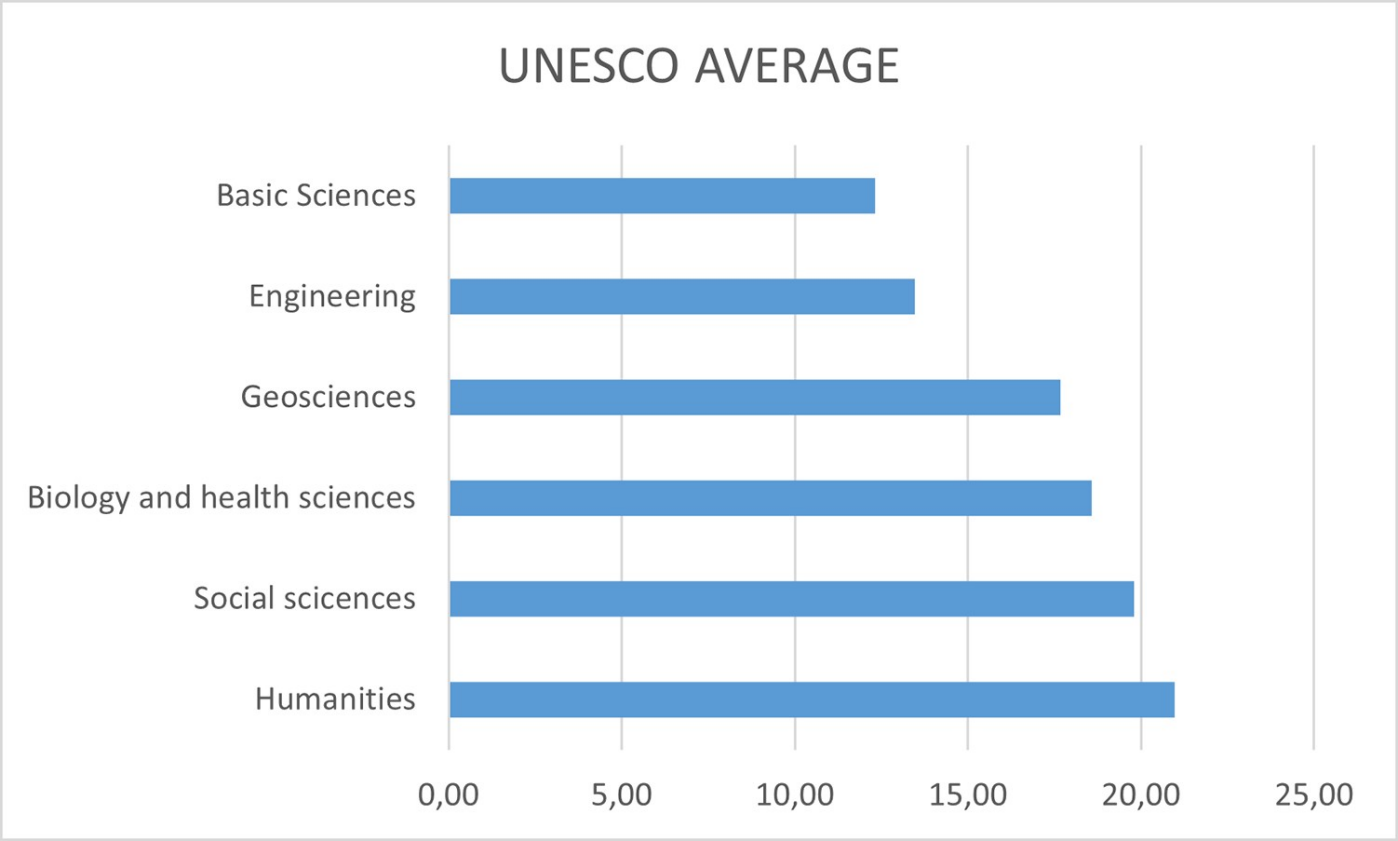
Non-inclusiveness indicator along the different UNESCO codes.

Fig 8 shows a visual representation of the non-inclusive indicator according to the UNESCO subject group displayed in Table 10, where the doctoral theses in Humanities achieve the greatest score, and other sciences as logic, mathematics, physics, chemistry or engineering obtain the best inclusive results. This fact makes sense with the typical topics under these subjects, commonly involving facts, numbers, formulae, etc.; social sciences and humanities might be prone to the use of words or opinions, where the inclusiveness might be affected. Although in the Humanities group, the field of Linguistics (9.90) obtains the lowest value of all (showing that in this field special attention is paid to the use of non-inclusive terms), the opposite occurs with the Law group, which obtains the highest value of all with 31.7:

**Table 10:** Age of the author, and impact on non-inclusiveness.

Group	Subject	Subject average	UNESCO average
Basic sciences	Logic	11.05	12.314
	Math	12.42	
	Astronomy	12.67	
	Physics	13.17	
	Chemistry	12.26	
Geosciences	Earth and Space	18.51	17.665
	Geography	18.51	
Biology and health sciences	Life sciences	18.71	18.573
	Medical sciences	17.39	
	Psychology	19.62	
Engineering	Agricultural sciences	14.46	13.46
	Technological sciences	12.46	
Social sciences	Anthropology	20.73	19.79
	Demography	21.23	
	Economical sciences	22.04	
	Legal sciences	31.70	
	Pedadogy	13.44	
	Political sciences	16.53	
	Sociology	12.86	
Humanities	History	26.58	20.97
	Linguistics	9.90	
	Art	24.14	
	Ethics	23.26	
	Philosophy	21.01	

**Fig 9:**
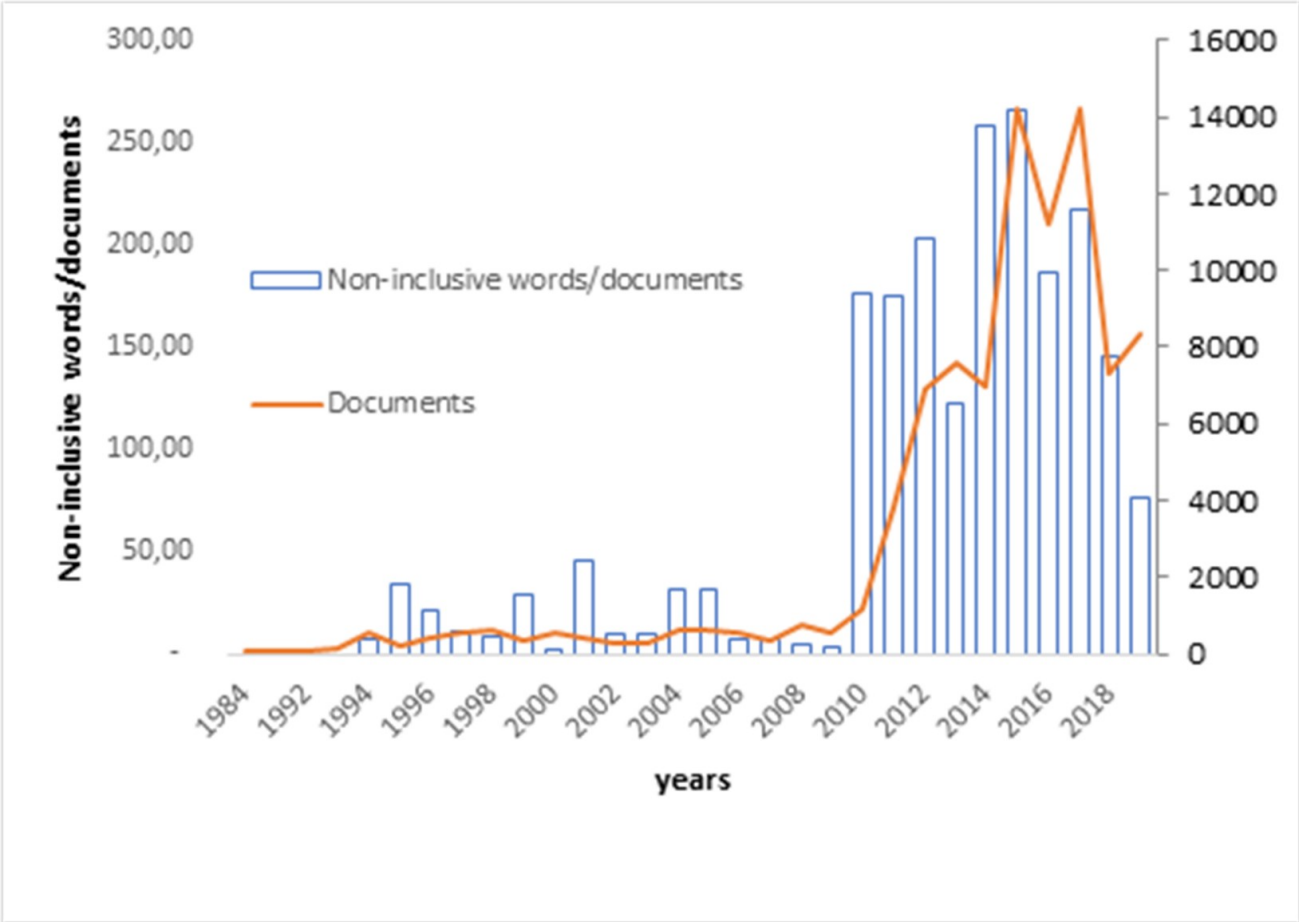
Comparative evolution of the non-inclusiveness and documents.

Fig 9 shows the evolution of the use of non-inclusive words by doctoral thesis over the years. It can be seen that, until 2010, doctoral theses maintained a steady level in the use of non-inclusive words. Since the publication in Spain of Royal Decree 99/2011, which regulates official doctoral education, there has been a very significant and continuous increase in the preparation and reading of theses. This increase also occurred in the use of non-inclusive terms, which could have a sociological behavioral explanation not considered in this study. In the last years (2018, 2019), there is a sharp decrease in the use of non-inclusive terms, which could be related to the current trend of avoiding gender discrimination. With the current values, the ratios prior to 2010 have not yet been reached.

Recalling the initial hypothesis enunciated in the introduction:

- H1: The development of an automatic text extraction algorithm would help to analyze gender bias.
- H2: The use of no-inclusive terms is directly related to age.
- H3: The use of no-inclusive terms is related to the field of knowledge.
- H4: Gender-bias is directly related to gender, being lower in women.
- H5: Unconscious bias is a characteristic of academic texts, particularly doctoral theses.

R1: The development of an automatic text extraction algorithm makes it possible to analyze gender bias in the case of doctoral theses written in Spanish. We understand that the system is applicable to documents outside the academic field, in the same language. The possibility of analyzing hundreds of thousands of texts with millions of words would become unapproachable otherwise.

R2: As can be seen in Table 9, the use of non-inclusive terms is directly related to age, increasing their use very deeply, assuming about 5.85 points above the average in the case of men and 4.25 in the case of women.

R3: Gender bias is grouped by areas with a behavior that could be considered logical. Where it appears least is in those documents with a use of scientific or mathematical terms. The areas where gender bias occurs most are those of Humanities and Social Sciences: with two extremes, the largest being the Laws subarea and the smallest being the Linguistics subarea.

R4: It has been shown that in the case of doctoral theses written in Spanish this is true in all cases and in all age ranges, also accentuating this male/female difference with age.

R5: The unconscious bias is characteristic of academic texts and, particularly, of doctoral theses. This has been shown to be true in the case of doctoral theses written in Spanish. That explains why the fields of engineering and basic sciences have the lowest values. If it was intentional, it would not depend on the scope and it would occur in any of them without a behavior pattern.

## Conclusion

The innovation provided by this research lies mainly in the ability to detect, within a digital text document in Spanish, whether the use of the language can be considered non-inclusive, based on a CNN that has been trained in the context of the doctoral thesis. A significant number of documents have been used, by means of all accessible doctoral theses from Spanish universities of the last 40 years, only manageable by data mining systems, so that the training allows identifying the terms within the context and effectively and compiling them in a novel dictionary of non-inclusive terms.

This research addresses the problem of providing an algorithm that can detect non-inclusive uses of the Spanish language without human supervision. It also shows that, although CNN usually requires a significant set of labeled data, when properly trained, it can show reasonable results in terms of accuracy, error, false positive and false negative rates.

As can be seen, there is a slight increasing trend in the use of non-inclusive terms as the doctoral student gets older, going from an average value of 13.68 to 21.18. Furthermore, for each age group, it is also observed that the number of non-inclusive occurrences is always higher in men than in women, concluding that there is a greater awareness of inclusiveness in women than in men, and also that this awareness grows as the candidate is younger. In terms of field of knowledge, the humanities are the most biased, discarding the subgroup of Linguistics, which has the least bias at all levels, and the field of science and engineering, which also have the least influence. This supports the assumption that the bias in academic texts (doctoral theses) is due to unconscious issues: otherwise, it would not depend on the field, age, gender, and it would occur in any field in the same ratio.

The results also allow us to study the evolution of inclusivity according to the author’s age, and the evolution over time. It is important to mention that new forms of inclusive language that included "wildcard" characters to abstract the word from being masculine or feminine (such as "todes", "tod@s", "tod*s" or "todxs") were explicitly searched for, but were not found in any document as part of the dissertation.

As for the limitations of this work, we can mention the following: this study is limited to a specific type of academic text, namely doctoral dissertations. However, the method of data collection and analysis could be extended to other types or to non-academic texts, and checked whether the most common non-inclusive terms match those obtained in this research. A proper training of the CNN on a different context of documents could provide the algorithm with adequate customization to address documents from different environments.

## References

[1] Hill, C.M., Kevin; Benson, Kathleen; Handley, Grace, Barriers and bias: The status of women in leadership. 2016, American Association of University Women AAUW. p. 66.

[2] Madsen S.R. and Andrade M.S., Unconscious Gender Bias: Implications for Women’s Leadership Development. 2018. 12(1): p. 62–67.

[3] (act/emp), B.f.E.A., Breaking barriers: unconscious gender bias in the workplace. 2017, International Labour Office.

[4] Mervis J., U.S. Study Shows Unconscious Gender Bias in Academic Science. Science, 2012. 337(6102): p. 1592. doi: 10.1126/science.337.6102.1592 23019620

[5] McKinsey_Company, Women in the workplace. 2020.

[6] (ACT/EMP), I.-B.f.E.A., Women in Business and Management: Gaining momentum. 2015.

[7] Bierema L.L, No women left behind: Critical leadership development to build gender consciousness and transform organizations, in Handbook of research on gender and leadership, Madsen S.R, Editor. 2017, Edward Elgar Publishing.: Cheltenham, England. p. 145–162.

[8] Diehl A.B. and Dzubinski L.M., Making the Invisible Visible: A Cross-Sector Analysis of Gender-Based Leadership Barriers. 2016. 27(2): p. 181–206.

[9] Emerson J., Don’t give up on unconscious bias training—Make it better. Harvard Business Review, 2017.

[10] Opoku A. and Williams N., Second-generation gender bias. International Journal of Ethics and Systems, 2019. 35(1): p. 2–23.

[11] EIGE, E.I.f.G.E., Toolkit on Gender-sensitive Communication. 2018, Vilnius, Lithuania: Publications Office of the European Union,.

[12] Association A., Publication manual of the American Psychological Association: the official guide to APA style. Seventh edition ed. 2020, Washington, D.C.: American Psychological Association.

[13] Teso E. and Crolley L., Gender-based linguistic reform in international organisations. Language Policy, 2013. 12(2): p. 139–158.

[14] Pauwels A., English. Spreading the feminist word: The case of the new courtesy title Ms in Australian English, in Gender Across Languages. 2001, John Benjamins.

[15] Romaine S., English. A corpus-based view of gender in British and American English, in Gender Across Languages. 2001, John Benjamins.

[16] Litosseliti L., Gender and language: Theory and practice. 2006.

[17] Hellinger M. and Bußmann H., Gender Across Languages: The linguistic representation of women and men. Volume 3. 2003: John Benjamins.

[18] Teso E., A comparative study of gender-based linguistic reform across four European countries. 2010, Liverpool John Moores University.: England.

[19] Mostafa M.M., More than words: Social networks’ text mining for consumer brand sentiments. Expert Systems with Applications, 2013. 40(10): p. 4241–4251.

[20] Khadjeh Nassirtoussi A., et al., Text mining for market prediction: A systematic review. Expert Systems with Applications, 2014. 41(16): p. 7653–7670.

[21] Gonzalez G.H., et al., Recent Advances and Emerging Applications in Text and Data Mining for Biomedical Discovery. Brief Bioinform, 2016. 17(1): p. 33–42. doi: 10.1093/bib/bbv087 26420781 PMC4719073

[22] Kumar B.S. and Ravi V., A survey of the applications of text mining in financial domain. Knowledge-Based Systems, 2016. 114: p. 128–147.

[23] Jagtap V., Analysis of different approaches to Sentence-Level Sentiment Classification. International Journal of Scientific Engineering and Technology (ISSN: 2277-1581), 2013. 2: p. 164–170.

[24] Singh, V.K., et al. Sentiment analysis of movie reviews: A new feature-based heuristic for aspect-level sentiment classification. in 2013 International Mutli-Conference on Automation, Computing, Communication, Control and Compressed Sensing (iMac4s). 2013.

[25] Tang D., et al., Learning Sentiment-Specific Word Embedding for Twitter Sentiment Classification. Vol. 1. 2014. 1555–1565.

[26] Castro J.L., et al., Extraction of fuzzy rules from support vector machines. Fuzzy Sets and Systems, 2007. 158(18): p. 2057–2077.

[27] Haddoud M., et al., Combining supervised term-weighting metrics for SVM text classification with extended term representation. Knowledge and Information Systems, 2016. 49(3): p. 909–931.

[28] Pratama, B.Y. and R. Sarno. Personality classification based on Twitter text using Naive Bayes, KNN and SVM. in 2015 International Conference on Data and Software Engineering (ICoDSE). 2015.

[29] Lin Y. and Wang J. Research on text classification based on SVM-KNN. in 2014 IEEE 5th International Conference on Software Engineering and Service Science. 2014.

[30] Trstenjak B., Mikac S., and Donko D., KNN with TF-IDF based Framework for Text Categorization. Procedia Engineering, 2014. 69: p. 1356–1364.

[31] Jiang L., et al., Deep feature weighting for naive Bayes and its application to text classification. Engineering Applications of Artificial Intelligence, 2016. 52: p. 26–39.

[32] Wang S., Jiang L., and Li C., Adapting naive Bayes tree for text classification. Knowledge and Information Systems, 2015. 44(1): p. 77–89.

[33] Zhang, H. and D. Li. Naïve Bayes Text Classifier. in 2007 IEEE International Conference on Granular Computing (GRC 2007). 2007.

[34] Zhang W. and Gao F., An Improvement to Naive Bayes for Text Classification. Procedia Engineering, 2011. 15: p. 2160–2164.

[35] Gadekallu T. R., Alazab M., Kaluri R., Maddikunta P. K. R., Bhattacharya S., Lakshmanna K., & Parimala M. (2021). Hand gesture classification using a novel CNN-crow search algorithm. Complex & Intelligent Systems, 1–14.

[36] Agarwal B. and Mittal N., Text Classification Using Machine Learning Methods-A Survey. 2014. p. 701–709.

[37] Allahyari M., et al., A Brief Survey of Text Mining: Classification, Clustering and Extraction Techniques. 2017.

[38] Jindal R., Malhotra R., and Jain A., Techniques for text classification: Literature review and current trends. Webology, 2015. 12.

[39] Zhang, X., J. Zhao, and Y. LeCun, Character-level convolutional networks for text classification, in Proceedings of the 28th International Conference on Neural Information Processing Systems—Volume 1. 2015, MIT Press: Montreal, Canada. p. 649–657.

[40] Lakshmanna K., & Khare N. (2016). Constraint-based measures for DNA sequence mining using group search optimization algorithm. International Journal of Intelligent Engineering & systems, 9, 91–100.

[41] Lakshmanna K., Kaluri R., Reddy G. T., Nagaraja G., & Subramanian D. V. (2016). An enhanced algorithm for frequent pattern mining from biological sequences. International Journal of Pharmacy and Technology, 8(2), 12776–12784.

[42] Kumar, M. E., Reddy, G. T., Sudheer, K., Reddy, M. P. K., Kaluri, R., Rajput, D. S., et al. (2017, November). Vehicle theft identification and intimation using gsm & iot. In IOP Conference Series: Materials Science and Engineering (Vol. 263, No. 4, p. 042062). IOP Publishing.

[43] Panigrahi R., Borah S., Bhoi A. K., Ijaz M. F., Pramanik M., Jhaveri R. H., et al. (2021). Performance Assessment of supervised classifiers for designing intrusion detection systems: A comprehensive review and recommendations for future research. Mathematics, 9(6), 690.

[44] Panigrahi R., Borah S., Bhoi A. K., Ijaz M. F., Pramanik M., Kumar Y., et al. (2021). A consolidated decision tree-based intrusion detection system for binary and multiclass imbalanced datasets. Mathematics, 9(7), 751.

[45] Prewitt-Freilino J., Caswell T.A., and Laakso E., The Gendering of Language: A Comparison of Gender Equality in Countries with Gendered, Natural Gender, and Genderless Languages. Sex Roles, 2011. 66.

[46] Stout J. and Dasgupta N., When He Doesn’t Mean You: Gender-Exclusive Language as Ostracism. Personality & social psychology bulletin, 2011. 37: p. 757–69. doi: 10.1177/0146167211406434 21558556

[47] Sarrasin O., Gabriel U., and Gygax P., Sexism and Attitudes Toward Gender-Neutral Language The Case of English, French, and German. Swiss Journal of Psychology, 2012. 71: p. 113–124.

[48] Gustafsson Sendén M., Renström E., and Lindqvist A., Introducing a gender-neutral pronoun in a natural gender language: The influence of time on attitudes and behavior. Frontiers in Psychology, 2015. 10.3389/fpsyg.2015.00893 PMC4486751 26191016

[49] Chen J.-Y. and Su J.-J., Differential Sensitivity to the Gender of a Person by English and Chinese Speakers. Journal of Psycholinguistic Research, 2011. 40(3): p. 195–203. doi: 10.1007/s10936-010-9164-9 21120608

[50] Dong Y., et al., Exploring the Cause of English Pronoun Gender Errors by Chinese Learners of English: Evidence from the Self-paced Reading Paradigm. Journal of Psycholinguistic Research, 2015. 44(6): p. 733–747. doi: 10.1007/s10936-014-9314-6 25178817

[51] Qiu L., et al., The role of gender information in pronoun resolution: evidence from Chinese. PLoS One, 2012. 7(5): p. e36156. doi: 10.1371/journal.pone.0036156 22615754 PMC3353965

[52] Formanowicz M., et al., Side Effects of Gender-Fair Language: How Feminine Job Titles Influence the Evaluation of Female Applicants. European Journal of Social Psychology, 2013. 43: p. 62–71.

[53] Cacciari C., Carreiras M., and Cionini C.B., When Words Have Two Genders: Anaphor Resolution for Italian Functionally Ambiguous Words. Journal of Memory and Language, 1997. 37(4): p. 517–532.

[54] Merkel E., Maass A., and Frommelt L., Shielding Women Against Status Loss:The Masculine Form and Its Alternatives in the Italian Language. Journal of Language and Social Psychology, 2012. 31(3): p. 311–320.

[55] Lévy A., Gygax P., and Gabriel U., Fostering the generic interpretation of grammatically masculine forms: When my aunt could be one of the mechanics. Journal of Cognitive Psychology, 2014. 26(1): p. 27–38.

[56] Calero Fernández M., Sexismo lingüístico. Análisis y propuestas ante la discriminación sexual en el lenguaje. 1999, Madrid: Editorial Narcea. 225.

[57] Calero Fernández M., El morfema género en el pensamiento de la Real Academia Española. ¿Cuestión que va más allá de la teoría gramatical? 2015. p. 447–474.

[58] Bosque, I. Sexismo lingüístico y visibilidad de la mujer. 2012; Available from: http://www.rae.es/sites/default/files/Sexismo_linguistico_y_visibilidad_de_la_mujer_0.pdf.

[59] García Meseguer Á., ¿Es sexista la lengua española?. Una investigación sobre el género gramatical. 1994, Barcelona (Spain): Editorial Paidós.

[60] Hartmann J., et al., Comparing automated text classification methods. International Journal of Research in Marketing, 2019. 36(1): p. 20–38.

[61] Orgeira P., et al., Decision Algorithm for the Automatic Determination of the Use of Non-Inclusive Terms in Academic Texts. Publications, 2020. 8: p. 41. doi: 10.1016/j.scitotenv.2018.01.219 29426134

[62] Yong-feng S. and Yan-ping Z., Comparison of text categorization algorithms. Wuhan University Journal of Natural Sciences, 2004. 9.

[63] Danesh A., Moshiri B., and Fatemi O., Improve text classification accuracy based on classifier fusion methods. 2007. 1–6.

[64] Basu A., Watters C., and Author M., Support Vector Machines for Text Categorization. 2003. 103.

[65] Gonen H. and Goldberg Y. Lipstick on a Pig: Debiasing Methods Cover up Systematic Gender Biases in Word Embeddings But do not Remove Them. in NAACL. 2019.

[66] Chakraborty T., Badie G., and Rudder B. Reducing gender bias in word embeddings. 2016.

[67] Bolukbasi, T., et al., Man is to computer programmer as woman is to homemaker? debiasing word embeddings, in Proceedings of the 30th International Conference on Neural Information Processing Systems. 2016, Curran Associates Inc.: Barcelona, Spain. p. 4356–4364.

[68] Dixon, L., et al., Measuring and Mitigating Unintended Bias in Text Classification, in Proceedings of the 2018 AAAI/ACM Conference on AI, Ethics, and Society. 2018, Association for Computing Machinery: New Orleans, LA, USA. p. 67–73.

[69] Nozza, D., C. Volpetti, and E. Fersini. Unintended Bias in Misogyny Detection. in 2019 IEEE/WIC/ACM International Conference on Web Intelligence (WI). 2019.

[70] Sharifirad S., Jafarpour B., and Matwin S., Boosting Text Classification Performance on Sexist Tweets by Text Augmentation and Text Generation Using a Combination of Knowledge Graphs. 2018. 107–114.

[71] Pitsilis G.K., Ramampiaro H., and Langseth H., Effective hate-speech detection in Twitter data using recurrent neural networks. Applied Intelligence, 2018. 48(12): p. 4730–4742.

[72] Mihalcea R. and Garimella A., What Men Say, What Women Hear: Finding Gender-Specific Meaning Shades. IEEE Intelligent Systems, 2016. 31(4): p. 62–67.

[73] Burger, J.D., et al., Discriminating gender on Twitter, in Proceedings of the Conference on Empirical Methods in Natural Language Processing. 2011, Association for Computational Linguistics: Edinburgh, United Kingdom. p. 1301–1309.

[74] Waseem Z. and Hovy D., Hateful Symbols or Hateful People? Predictive Features for Hate Speech Detection on Twitter. 2016. 88–93.

[75] Badjatiya, P., M. Gupta, and V. Varma, Stereotypical Bias Removal for Hate Speech Detection Task using Knowledge-based Generalizations, in The World Wide Web Conference. 2019, Association for Computing Machinery: San Francisco, CA, USA. p. 49–59.

[76] Davidson T., et al., Automated Hate Speech Detection and the Problem of Offensive Language. 2017.

[77] Del Vigna F., et al., Hate me, hate me not: Hate speech detection on Facebook. 2017.

[78] Teso E., et al., Application of text mining techniques to the analysis of discourse in eWOM communications from a gender perspective. Technological Forecasting and Social Change, 2018. 129: p. 131–142.

[79] Basta, C., M. Costa-jussa, and N. Casas. Evaluating the Underlying Gender Bias in Contextualized Word Embeddings. in First Workshop on Gender Bias in Natural Language Processing. 2019. Florence, Italy: Association for Computational Linguistics.

[80] Hitti Y., et al. Proposed Taxonomy for Gender Bias in Text; A Filtering Methodology for the Gender Generalization Subtype. 2019.

[81] May, C., et al. On Measuring Social Biases in Sentence Encoders. in Proceedings of the 2019 Conference of the North American Chapter of the Association for Computational Linguistics: Human Languages Technologies. 2019. Minneapolis, Minnesota: Association for Computational Linguistics.

[82] Zhou, P., et al. Examining Gender Bias in Languages with Grammatical Gender. in 2019 Conference on Empirical Methods in Natural Language Processing and the 9th International Joint Conference on Natural Language Processing (EMNLP-IJCNLP). 2019. Hong Kong, China: Association for Computational Linguistics.

[83] Zhao, J., et al. Learning Gender-Neutral Word Embeddings. in Conference on Empirical Methods in Natural Language Processing. 2018. Brussels, Belgium: ssociation for Computational Linguistics.

[84] Bordia, S. and S. Bowman. Identifying and Reducing Gender Bias in Word-Level Language Models. in NAACL HLT 2019–2019 Conference of the North American Chapter of the Association for Computational Linguistics: Human Language Technologies—Proceedings of the Student Research Workshop. 2019. Association for Computational Linguistics (ACL).

[85] Leavy, S. Gender Bias in Artificial Intelligence: The Need for Diversity and Gender Theory in Machine Learning. in 2018 IEEE/ACM 1st International Workshop on Gender Equality in Software Engineering (GE). 2018.

[86] Diakopoulos N., Accountability in algorithmic decision making. Commun. ACM, 2016. 59(2): p. 56–62.

[87] Ortigosa A., Martín J.M., and Carro R.M., Sentiment analysis in Facebook and its application to e-learning. Computers in Human Behavior, 2014. 31: p. 527–541.

[88] Kim, Y., Convolutional Neural Networks for Sentence Classification. Proceedings of the 2014 Conference on Empirical Methods in Natural Language Processing, 2014. 10.18653/v1/d16-1076 PMC5300751 28191551

[89] Lever J., Krzywinski M., and Altman N., Classification evaluation. Nature Methods, 2016. 13(8): p. 603–604.

[90] Pesce A. and Etchezahar E., Actitudes y Uso del Lenguaje Inclusivo según el Género y la Edad. Búsqueda, 2019. 6(23): p. 472.

[91] Gustafsson Sendén M., Bäck E.A., and Lindqvist A., Introducing a gender-neutral pronoun in a natural gender language: the influence of time on attitudes and behavior. 2015. 6(893). 10.3389/fpsyg.2015.00893 PMC4486751 26191016

[92] Sczesny S., Moser F., and Wood W., Beyond Sexist Beliefs:How Do People Decide to Use Gender-Inclusive Language? 2015. 41(7): p. 943–954. 10.1177/0146167215585727 26015331

[93] Douglas K.M. and Sutton R.M., “A Giant Leap for Mankind” But What About Women? The Role of System-Justifying Ideologies in Predicting Attitudes Toward Sexist Language. 2014. 33(6): p. 667–680.

[94] Parks J.B. and Roberton M.A., Generation Gaps in Attitudes Toward Sexist/Nonsexist Language. 2008. 27(3): p. 276–283.

[95] Basile V., et al. SemEval-2019 Task 5: Multilingual Detection of Hate Speech Against Immigrants and Women in Twitter. in SemEval@NAACL-HLT. 2019.

